# Nucleosomes accelerate transcription factor dissociation

**DOI:** 10.1093/nar/gkt1319

**Published:** 2013-12-17

**Authors:** Yi Luo, Justin A. North, Sean D. Rose, Michael G. Poirier

**Affiliations:** ^1^Biophysics Graduate Program, The Ohio State University, Columbus, OH 43210, USA, ^2^Department of Physics, The Ohio State University, Columbus, OH 43210, USA and ^3^Department of Chemistry and Biochemistry, The Ohio State University, Columbus, OH 43210, USA

## Abstract

Transcription factors (TF) bind DNA-target sites within promoters to activate gene expression. TFs target their DNA-recognition sequences with high specificity by binding with resident times of up to hours *in vitro*. However, *in vivo* TFs can exchange on the order of seconds. The factors that regulate TF dynamics *in vivo* and increase dissociation rates by orders of magnitude are not known. We investigated TF binding and dissociation dynamics at their recognition sequence within duplex DNA, single nucleosomes and short nucleosome arrays with single molecule total internal reflection fluorescence (smTIRF) microscopy. We find that the rate of TF dissociation from its site within either nucleosomes or nucleosome arrays is increased by 1000-fold relative to duplex DNA. Our results suggest that TF binding within chromatin could be responsible for the dramatic increase in TF exchange *in vivo*. Furthermore, these studies demonstrate that nucleosomes regulate DNA–protein interactions not only by preventing DNA–protein binding but by dramatically increasing the dissociation rate of protein complexes from their DNA-binding sites.

## INTRODUCTION

Initiation of eukaryotic gene expression involves transcription factor (TF) binding to DNA-target sites at gene promoters within chromatin (1,2). Chromatin is comprised of a long array of nucleosomes, each containing ∼147 bp of DNA wrapped around a histone protein octamer core (3,4). TF-target sequences are often located near the DNA entry–exit region of the nucleosome (5–8), so that the nucleosome structure sterically occludes TF occupancy at its binding site. However, transient partial unwrapping fluctuations in addition to chromatin remodeling (9) and nucleosome disassembly (10) provide limited access to TF-target sites (11,12).

**Figure 1.**
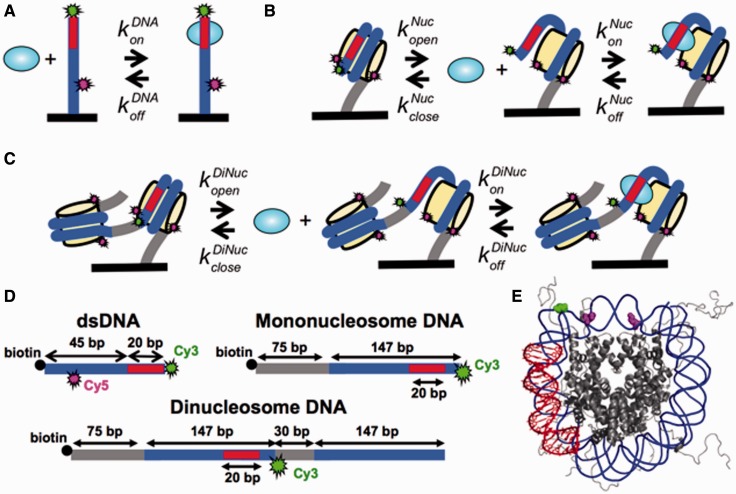
DNA and nucleosome constructs. Kinetic models of TF binding to (A) DNA, (B) single nucleosomes and (C) nucleosome arrays. (D) DNA constructs for single molecule TIRF measurements with Cy3 (green), Cy5 (magenta), biotin (black circle), 601 NPS (blue) and a Gal4- or LexA-target sequence (red). DNA molecules for making mononucleosome and dinucleosome array were labeled with Cy3 fluorophore as the FRET donor. DNA molecules for PIFE experiments were labeled with Cy3 as the PIFE indicator and Cy5 to help locate the molecule during single-molecule experiments. (E) Structure of the nucleosome (PDB: 1KX5) that indicates the location of the TF-target sequence (red), the Cy3 fluorophore location (green) and the Cy5 fluorophore location (magenta).

TFs target specific genes by binding particular DNA sequences with high affinities that are quantified by the dissociation constant, *K_D_* = *k*_off_/*k*_on_ (Figure 1A). The *K_D_* is the characteristic concentration for binding and can be determined experimentally by measuring *S*_0.5_, the concentration of TF at which 50% of the target DNA sequence is bound. Under conditions where the DNA-target sequence concentration is significantly below the *K_D_*, *S*_0.5_ = *K_D_*. The *K_D_* is typically between nanomolar and picomolar for DNA-binding TFs. This is usually achieved by having relatively long resident times on, and slow dissociation rates from the target sequence. *In vitro*, TFs can have residence times of about an hour at their DNA-recognition sequence (13,14), which implies dissociation rates as low as 10^−^^4^s^−1^. Surprisingly, *in vivo* fluorescence recovery after photobleaching measurements of TF dynamics find that TFs exchange on the scale of seconds (15–19) even though their resident times at their DNA-target site *in vitro* are much longer. The mechanisms by which TF dissociation is dramatically accelerated remain unknown (20,21).

Previous studies have investigated the influence of nucleosomes on site-specific DNA-binding proteins such as TFs. Restriction enzyme (RE) cleavage experiments have demonstrated that target sites within nucleosomes are accessible to protein binding by partial DNA unwrapping from the histone octamer (HO) core (12,22). Fluorescence resonance energy transfer (FRET) measurements of nucleosome unwrapping were then used to detect binding of a model TF, LexA, to its target sequence between the 8^th^ and 27^th^ base pairs of the nucleosome under low ionic conditions (11,23). They found that LexA bound to its target sequence within a partially unwrapped nucleosome, albeit the concentration to bind 50% of the nucleosomes, *S*_0.5_, was 100-fold higher relative to duplex DNA. More recently, FRET measurements determined that monovalent ionic conditions, DNA sequence and histone post-translational modifications influenced LexA occupancy (24–26). Previous studies have inferred the site exposure equilibrium constant from the ratio of the *S*_0.5_ for LexA binding to duplex DNA relative to binding to nucleosomes. This assumed that the increase in *S*_0.5_ is due to sterically occluding TF and RE binding from their binding sites and that the dissociation rate of the TF and RE are the same between duplex DNA and partially unwrapped nucleosomal DNA.

We investigated the hypothesis that the nucleosome not only suppresses TF binding but also influences TF dissociation. The dynamics of TF binding to and dissociation from its site within duplex DNA, single nucleosomes and nucleosome arrays were quantified using single molecule total internal reflection fluorescence (smTIRF) microscopy (27). We detected binding of two TFs, LexA and Gal4, to their target sites within duplex DNA with Protein Induced Fluorescence Enhancement (PIFE; Figure 1A and D) (28). PIFE detects protein binding by attaching a fluorophore such as Cy3 adjacent to the TF-target sequence. Upon TF binding, the fluorescence emission increases. We quantified fluctuations in Cy3 fluorescence to determine the binding and dissociation rates with duplex DNA. We then separately detected TF binding to its target site within mono- and dinucleosomes with FRET (Figure 1B, C and D) (29). TF binding traps the nucleosome in a partially unwrapped state. This increases the distance between the Cy3 and Cy5 fluorophores in the nucleosome and results in a reduction in FRET efficiency. By monitoring fluctuations between high and low FRET efficiency, we determined the binding and dissociation rates of both LexA and Gal4 within nucleosomes. We find that nucleosomes not only suppress TF binding, but enhance the rate of dissociation by up to three orders of magnitude. These measurements indicate that nucleosomes regulate TF occupancy not only by blocking binding but by increasing the dissociation rate. Furthermore, our results indicate that nucleosomes can facilitate TF exchange and is a potential mechanism for the measured difference in the rate of TF dissociation from chromatin *in vivo* and from duplex DNA *in vitro*.

## MATERIALS AND METHODS

### Preparation of TFs

LexA protein was expressed and purified from pJWL288 plasmid (generous gift from Dr Jonathan Widom) as previously described (30). Briefly, LexA was expressed in *Escherichia coli* BL21(DE3)pLysS cells (Invitrogen) by inducing with 0.2 mM IPTG for 2 h. Cells were harvested by centrifugation and resuspended at 50 ml per 1 l starting culture in Buffer A (50 mM Tris–HCl pH 8.0, 200 mM NaCl, 1 mM DTT, 0.5 mM EDTA, 10% w/v sucrose). The cells were lysed by lysozyme and cell debris were removed by centrifugation. DNA was removed by precipitation with 35% polyethyleneimine and then LexA was precipitated twice by 40% ammonium sulfate. LexA was resuspended in Buffer B (20 mM potassium phosphate pH 7.0, 0.5 mM EDTA, 10% v/v glycerol) with 1 mM DTT and 500 mM NaCl, and then dialyzed against the same buffer overnight. Dialyzed LexA was diluted 2.5-fold with Buffer B plus 1 mM DTT to give a final NaCl concentration of 200 mM before loading onto a cellulose phosphate column. LexA was then eluted by a linear gradient of Buffer B from 200 mM to 800 mM NaCl. Fractions containing LexA were then loaded onto a hydroxyapatite column and then eluted with a gradient of Buffer C (10% v/v glycerol plus desired concentration of potassium phosphate pH 7.0) from 50 mM to 400 mM potassium phosphate. Fractions containing high purity LexA were then dialyzed extensively against Buffer D (10 mM PIPES–NaOH pH 7.0, 0.1 mM EDTA, 10% v/v glycerol, 200 mM NaCl) and stored at –80°C.

The Gal4 expression vector was prepared by cloning the Gal4 gene for residues 1–147 from *Saccharomyces cerevisiae* genomic DNA (generous gift from Dr Yvonne Fondufe-Mittendorf) into pET3a at the NdeI and BamHI sites. Gal4(1–147) was expressed in *E. coli* Rosetta(DE3)pLysS cells (Millipore) by inducing with 1 mM IPTG for 3 h. Cells were harvested by centrifugation and resuspended at 50 ml per 1 l starting culture in Buffer A [50 mM Tris pH 8.0, 200 mM NaCl, 1 mM DTT, 10 μM ZnCl_2_, 1 mM phenylmethanesulfonyl fluoride (PMSF)] with 20 μg/ml leupeptin, and 20 μg/ml pepstatin. The cells were lysed by sonication and cell debris were removed by centrifugation. DNA was removed by precipitation with 35% polyethyleneimine and then Gal4(1–147) was precipitated by 40% ammonium sulfate (5). Gal4(1–147) was resuspended in Buffer A with 20 μg/ml leupeptin and 20 μg/ml pepstatin and loaded onto a Sephacryl 200HR gel filtration column (GE healthcare) (1). Fractions containing Gal4(1–147) were dialyzed into Buffer B (20 mM potassium phosphate pH 7.0, 10% glycerol, 1 mM DTT, 10 μM ZnCl_2_, 1 mM PMSF) with 200 mM NaCl, directly loaded onto a cellulose phosphate column and then eluted by linear gradient of Buffer B from 200 mM NaCl to 800 mM NaCl. Fractions containing Gal4(1–147) were dialyzed into Buffer C (25 mM Tris pH 7.5, 1 mM DTT, 10 μM ZnCl_2_, 1 mM PMSF) with 200 mM NaCl and loaded directly onto a TSKgel SP5-PW (Tosoh biosciences) anion exchange column, and eluted by a linear gradient of Buffer C with 200 mM NaCl to 800 mM NaCl. Fractions containing high purity Gal4(1–147) were dialyzed into Buffer D (10 mM HEPES pH 7.5, 200 mM NaCl, 10% glycerol, 1 mM DTT, 10 μM ZnCl_2_, 1 mM PMSF) and stored at –80°C

### Preparation of DNA molecules

DNA molecules for PIFE experiments and DNA molecules for making mononucleosome and dinucleosome array (Figure 1D) were prepared by PCR with Cy3/Cy5/biotin-labeled oligonucleotides from plasmid containing the 601 nucleosome positioning sequence (NPS) with a consensus LexA-binding site (TACTGTATGAGCATACAGTA) or Gal4-binding site (CCGGAGGACTGTCCTCCGG) at bases 8–27 (LexA) or bases 8–26 (Gal4). Oligonucleotides (Supplementary Table S1) were labeled with Cy3 or Cy5 NHS ester (GE healthcare) at an amino group attached at the 5′-end or to a modified internal thymine and then HPLC purified on a 218TP C18 column (Grace/Vydac). Following PCR amplification, each DNA molecule was purified by HPLC on a Gen-Pak Fax column (Waters).

The dinucleosome DNA was synthesized by ligation of two shorter PCR products. PCR synthesized DNA molecules containing a TspRI site and a 601 sequence or 601 sequence with a LexA-binding site were digested by TspRI in NEB buffer #4 (New England Biolabs). Digestion products were purified by polyacrylamide gel electrophoresis. Purified two short DNA pieces with TspRI sticky ends were mixed and ligated with T4 ligase (New England Biolabs) in supplied buffer plus 2 mM ATP and HPLC purified with a Gen-Pak Fax column to remove unligated fragments.

### Preparation of HOs

*Xenopus laevis* recombinant histones were expressed and purified as previously described (31). Plasmids encoding histones H2A(K119C), H2B, H3 and H4 were generous gifts from Dr Karolin Luger (Colorado State University) and Dr Jonathan Widom. Mutation H3(C110A) was introduced by site-directed mutagenesis (Stratagene). Each of the four histones were combined at equal molar ratios, refolded and purified as previously described (31). H2A(K119C)-containing HO was labeled with Cy5-maleamide (GE Healthcare) as previously described (25).

### Preparation of nucleosomes

Nucleosomes were reconstituted from Cy3-labeled DNA and purified Cy5-labeled HO by salt double dialysis and purified by sucrose gradient as previously described (25). Mononucleosome reconstitutions contained a molar ratio of 0.85:1 of HO : DNA; dinucleosome reconstitutions contained a mass ratio of 1.3 : 1 : 2 of HO : template DNA : lambda DNA. HO and DNA were mixed in 0.5× TE pH 8.0 with 1 mM Benzamidine hydrochloride (BZA) and 2 M NaCl in a volume of 50 µl and then loaded into a dialysis chamber. The small dialysis chamber was then placed into a large dialysis tube containing 80 ml of 0.5× TE pH 8.0 with 1 mM BZA and 2 M NaCl and then dialyzed extensively against 0.5× TE pH 8.0 with 1 mM BZA at 4°C. Dialyzed nucleosomes were loaded onto sucrose gradient and purified by centrifugation on an Optima L-90 K Ultracentrifuge (Beckman Coulter) with a SW41 rotor. Mononucleosomes were purified by a gradient of sucrose from 5% to 30% w/v and dinucleosome arrays were purified by a gradient of sucrose from 5% to 35% w/v. Fractions containing nucleosomes were then collected and concentrated.

### Electromobility shift assays

DNA containing the LexA-target site was incubated at 0.2 nM with 0–100 nM LexA in 0.5× TE pH 8.0 for 2 min at 20°C and then resolved by Electrophoretic Mobility Shift Assay (EMSA) with a native 5% polyacrylamide gel in 0.3× TBE. DNA containing the Gal4-target site was incubated at 0.2 nM with 0–3 nM Gal4 in 10 mM Tris–HCl pH 8.0, 130 mM NaCl, 0.1 mg/ml BSA, 10% glycerol, 0.005% TWEEN20, 1 mM DTT and 5 ng/μl poly-dIdC (Sigma P4929) for 2 min at 20°C and then resolved by EMSA with a native 5% polyacrylamide gel in 0.3× TTE.

### Ensemble PIFE measurements

TF binding to its target site within Cy3-labeled DNA was determined by PIFE (28), where the Cy3 fluorescence increases upon protein binding. Fluorescence spectra during TF titrations were acquired with a Fluoromax4 (Horiba) using an excitation of 510 nm. LexA titrations were done with 0.2 nM fluorophore-labeled DNA in 10 mM Tris–HCl pH 8.0, 130 mM NaCl, 10% Glycerol, 0.005% TWEEN20, 0.1 mg/ml BSA and 1% BME. Gal4 titrations were done with 0.1 nM fluorophore-labeled DNA in 10 mM Tris–HCl pH 8.0, 130 mM NaCl, 10% Glycerol, 0.005% TWEEN20 and 1% BME. Fluorescence spectrums were analyzed with Origin (OriginLab) to determine the change in Cy3 fluorescence. We carried out PIFE measurements of LexA and Gal4 titrations with dsDNA that did not contain their respective target sequences (Supplementary Figure S1A and B) and did not observe an increase in PIFE. This implies that the increase in PIFE is due to LexA and Gal4 binding to their target sequences.

### Ensemble FRET measurements

TF binding and nucleosome site accessibility equilibrium constants were measured with LexA binding to its target site buried within Cy3–Cy5-labeled nucleosomes as previously described (11,25). TF binding to its target site traps the nucleosome into a partially unwrapped state (11) resulting in a partial reduction in FRET efficiency, which we used to detect TF binding. LexA titrations were done with 5 nM nucleosomes in 50 mM HEPES pH 7.5, 130 mM NaCl, 10% Glycerol, 0.005% TWEEN20, 0.1 mg/ml BSA, 2 mM Trolox (Sigma 238813), 0.0115% v/v Cyclooctatetraene (COT, Sigma 138924) and 0.012% v/v 3-Nitrobenzyl alcohol (NBA, Sigma 146056). Gal4 titrations were done with 0.2 nM nucleosomes in 10 mM Tris–HCl pH 8.0, 130 mM NaCl, 10% Glycerol and 0.005% TWEEN20. FRET efficiency measurements were determine by the (ratio)_A_ method (29). Fluorescence emission spectra were measured as previously described (25). We previously determined that nonspecific DNA binding of LexA does not reduce the FRET efficiency and that binding of LexA to its target sequence within the nucleosome does not induce dissociation of H2A–H2B heterodimers (25). We also carried out control titrations of Gal4 with nucleosomes that do not contain the Gal4-binding site (Supplementary Figure S1C). As with LexA, non-specific binding of Gal4 did not reduce the FRET efficiency. These control measurements imply that our observed reduction in FRET is due to TF binding to their target sequence.

### Single molecule smTIRF microscope

The smTIRF microscope was built on an IX71-inverted microscope (Olympus) as previously described (27). 532 and 638 nm diode lasers (Crystal Lasers) were used for Cy3 and Cy5 excitation. The excitation beams were expanded and then focused through a quartz prism (Melles Griot) at the surface of the quartz flow cell. A 1.2 N.A. water immersion objective (Olympus) was used to collect the Cy3 and Cy5 fluorescence which were separately imaged onto a PhotonMax EMCCD camera (Princeton Instruments) with a Dualview (Optical Insights) containing bandpass filters and a dichroic beam splitter (Chroma Tech). Each image time series was acquired with a PC using Winview (Roper Scientific) and analyzed as describe below.

### Flow cell preparation

Quartz microscope slides (G. Finkenbeiner) were functionalized with poly-ethylene glycol (PEG, Laysan Bio, MPEG-SVA-5000) and biotin-PEG (Laysan Bio, Biotin-PEG-SVA-5000) and assembled with glass coverslips to make the flow cell. Briefly, quartz microscope slides and glass coverslips were cleaned in toluene and ethanol with sonication, and then further cleaned in Piranha solution (3 : 1 mixture of concentrated sulfuric acid to 50% hydrogen peroxide) and washed in 1 M sodium hydroxide. The cleaned slides were treated with 2% v/v 3-aminopropyl-triethoxysilane (MP biomedicals 215476680) in acetone, and then with 10% w/v PEG in 0.1 M potassium tetraborate pH8.1 (100 : 1 mass ratio mixture of mono-functional PEG to biotin-PEG). Functionalized quartz slides and coverslips were assembled into microscope flow cells using parafilm with cut channels. Before each experiment, the flow cell is treated sequentially with 1 mg/ml BSA, 20 µg/ml streptavidin and biotin-labeled DNA/nucleosome samples to form surface tethers.

### Single molecule fluorescence measurements of TF binding and dissociation

Biotinylated sample molecules (DNA, mononucleosome or dinucleosomes arrays) were allowed to incubate in the flow cell at room temperature for 5 min and then washed out with imaging buffer containing the desired concentration of TF protein. The samples were first exposed to 638 nm excitation to determine which molecules contained Cy5, then the times of Cy3 and Cy5 emission was acquired while exciting with 532 nm. The imaging buffer for nucleosomes contained 50 mM HEPES pH 7.5, 130 mM NaCl, 10% v/v glycerol, 0.005% v/v TWEEN20, 0.1 mg/ml BSA, 2 mM Trolox, 0.0115% v/v COT, 0.012% v/v NBA, while the imaging buffer for duplex DNA contained 10 mM Tris–HCl pH 8.0, 130 mM NaCl, 10% v/v glycerol, 0.005% v/v TWEEN20, 0.1 mg/ml BSA, 1% v/v BME. An oxygen-scavenging system containing 1.6% w/v glucose, 450 µg/ml glucose oxidase (Sigma G2133) and 22 µg/ml catalase (Sigma C3155) was also supplied with the imaging buffer to suppress photobleaching of the fluorophores (2).

Single-molecule time series were fit to two-state step function by hidden Markov method using vbFRET Matlab program (13) provided by Dr Ruben Gonzalez (Columbia University). Idealized time series were further analyzed using custom written Matlab programs to determine the dwell-time distributions of the TF bound and unbound states. The FRET efficiency from 70% of the mononucleosomes and 70% of the dinucleosomes fluctuated in the presence of TF. We included each of these time traces for determining the bound and unbound dwell-time distribution with nucleosomes. The Cy3 fluorescence of 25% of DNA contained PIFE fluctuations in the presence of LexA. We included the analysis of the times series of all of the molecules with PIFE fluctuations for determining the bounded and unbounded dwell-time distributions with DNA. Weighted fits of the dwell-time distributions to single exponential-decay curves were used to obtain the characteristic times and rate constants for the transition between bounded and unbounded states.

## RESULTS

### LexA has a resident time of ∼5 min at its target sequence within duplex DNA

**Figure 2.**
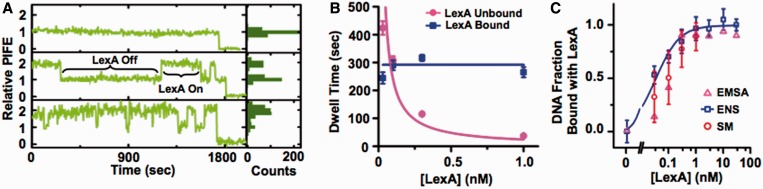
Single molecule measurements of LexA binding and dissociation to DNA. (A) Single molecule PIFE traces of LexA binding to its target site in Cy3-labeled duplex DNA with 0 (top), 0.1 (middle) and 1 (bottom) nM LexA. The histogram on the right shows the distribution of Cy3 fluorescence for each trace. (B) The unbound (magenta circles) and bound (blue squares) dwell times, *τ*_unbound_, with duplex DNA as a function of LexA concentration. Each dwell time was determined from an exponential fit to the dwell-time histogram (Supplementary Figure S2). The LexA concentration dependence of the unbound dwell times were fit to *τ*_unbound_ = *A*/[LexA] with *A* = (20 ± 6) s nM, and the bound dwell times were fit to *τ*_bound_ = constant = (290 ± 20) s, respectively. (C) The fraction of DNA bound by LexA as determined by EMSA (magenta triangles), ensemble PIFE measurements fit with a non-cooperative binding curve with a *K_D_* = (0.13 ± 0.06) nM (blue squares) and single molecule PIFE measurements (red circles).

We first investigated the binding dynamics of LexA, a TF that binds as a homodimer, as do many high affinity TFs (32). LexA has been extensively studied with DNA (33,34) and as a model TF that binds within nucleosomes (5,11,23,25,26,35). We did single-molecule PIFE experiments to study the binding of LexA to its target sequence within duplex DNA. A Cy3 fluorophore was attached adjacent to the LexA- target sequence. The fluorescence of Cy3 is enhanced upon LexA binding (Figure 2A). The enhancement is sensitive only to LexA binding to the specific recognition sequence (Supplementary Figure S1A). To confirm that the Cy3 signal acquired was from immobilized DNA molecules instead of any possible background fluorescence in the flow cell, a Cy5 fluorophore was also attached on the same DNA further away from the LexA-target site (Figure 1D) so that only the signal from a Cy3 that co-localized with a Cy5 was selected for analysis. From >333 molecules, 2000-s time series of Cy3 fluorescence were acquired at each LexA concentration (0.03, 0.1, 0.3 and 1 nM). The Cy3 fluorescence enhancement upon LexA binding allows us to determine the dwell time of each bounded and unbounded DNA state. The bounded and unbounded dwell-time histograms were determined for each LexA concentration (Supplementary Figure S2), and fit to a single exponential decay, *A*exp(–*t*/*τ*), where *τ* is the characteristic decay time. The exponential decay fits of the bounded and unbounded dwell-time histograms determine the characteristic bound time, *τ*_bound,_ and unbound time, *τ*_unbound_, respectively. The *τ*_bound_ is constant for increasing concentrations of LexA with a value of (290 ± 20) s, implying that *k*_off_ = 1/*τ*_bound_ = (3.4 ± 0.2) × 10^−^^3^s^−1^. The *τ*_unbound_ decreases as *A*/[LexA], where 1/*A* = *k*_on_ = (0.05 ± 0.01)s^−1^ nM^−1^ (Figure 2B). This yields a dissociation constant, *K_D_* = *k*_off_/*k*_on_ = (0.07 ± 0.02) nM (Table 1). This *K_D_* is consistent with ensemble measurements of *S*_0.5_ by EMSA and by ensemble PIFE measurements (Figure 2C, Supplementary Figure S3A). Furthermore, we determined the fraction of time the DNA molecules were bound by LexA during the smTIRF measurements and plotted this versus LexA concentration. These values agreed with the EMSA and ensemble PIFE measurements of LexA binding to DNA (Figure 2C). This agreement of ensemble and smTIRF measurements with duplex DNA indicates that the surface tethering does not impact the TF binding and dissociation dynamics.

**Table 1.** Binding and dissociation time constant and rate constant obtained from real-time single-molecule experiments

	*τ*_bound_	*τ*_unbound_	*k*_off_	*k*_on_	*K_D_*
	(s)	(s nM)	(s^−1^)	(s^−1^ nM^−1^)	(nM)
LexA–DNA	290 ± 20	20 ± 6	(3.4 ± 0.2) × 10^–3^	0.05 ± 0.01	0.07 ± 0.02
LexA–monoNuc	0.31 ± 0.05	(1.1 ± 0.3) × 10^–4^	3.3 ± 0.6	(9 ± 2) × 10^–5^	(4 ± 2) × 10^4^
LexA–diNuc	0.29 ± 0.05	(1.1 ± 0.3) × 10^–4^	3.5 ± 0.3	(9 ± 2) × 10^–5^	(4 ± 1) × 10^4^
Gal4–DNA	≫2000	ND	≪(5 × 10^–4^)	ND	ND
Gal4–monoNuc	50 ± 2	2.5 ± 0.1	0.020 ± 0.001	0.40 ± 0.02	0.051 ± 0.005

ND, indicates not determined.

### LexA has a resident time of ∼0.3 s at its target sequence within the nucleosome entry–exit region

**Figure 3.**
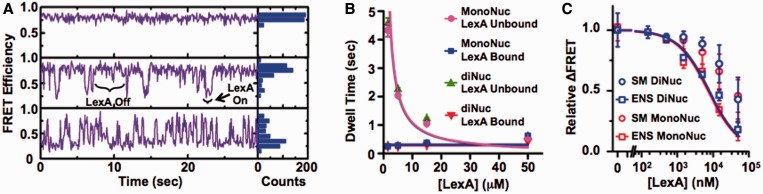
Single molecule measurements of LexA binding and dissociation to single nucleosomes and dinucleosome arrays. (A) Single molecule FRET traces of LexA-trapping nucleosomes in partially unwrapped states with 0 (top), 5 (middle) and 50 (bottom) µM LexA. The histogram shows the distribution of the FRET for each trace. (B) The unbound (magenta circles) and bound (blue squares) dwell times with mononucleosomes, and the unbound (green triangles) and bound (red inverted triangles) dwell times with dinucleosome arrays as a function of LexA concentration. Each dwell time was determined from an exponential fit to the dwell time histogram (Supplementary Figure S5 and S6). The LexA concentration dependence of the unbound dwell times were fit to *τ*_unbound_ = *A*/[LexA] with *A*_monoNuc_ = (1.1 ± 0.3) × 10^–4 ^s nM and *A*_diNuc_ = (1.1 ± 0.3) × 10^–4 ^s nM, and the bound dwell times were fit to *τ*_bound_ = constant with *τ*_bound(monoNuc)_ = (0.31 ± 0.05) s and *τ*_bound(diNuc)_ = (0.29 ± 0.05) s. (C) The relative change in energy transfer efficiency versus the LexA concentration determined by both ensemble and single molecule measurements. The ensemble measurements relied on analysis of fluorescence spectra (Supplementary Figure S4) by the (ratio)_A_ method with mononucleosomes (red squares) and dinucleosome (blue squares). The fraction of time in the low- and high-FRET states were determined from single-molecule FRET time series for both mononucleosomes (red circles) and dinucleosomes (blue circles).

To determine the influence of the nucleosome on TF binding and dissociation dynamics, we investigated with smTIRF LexA binding and dissociation at its target site located between the 8-th and the 27-th bp of sucrose gradient purified nucleosomes (Supplementary Figure S3C). LexA binding to its target site traps the nucleosome in a partially unwrapped state causing a significant drop in FRET efficiency (11). The nucleosome is labeled on the 5′-end with Cy3, while H2A(K119C) is labeled with Cy5. One of the two Cy5 fluorophores is within the Förster radius of the Cy3 molecule for fully wrapped nucleosome resulting in a high-FRET efficiency. Upon LexA binding, which traps the nucleosomes in a partially unwrapped state, the distance between the Cy3 molecule and the nearby Cy5 molecule will increase and result in a reduction in FRET efficiency. While there are two Cy5 fluorophores in the nucleosome, the FRET efficiency can still be estimated by the ratio_A_ method (11). Nucleosomes spontaneously partially unwrap and rewrap (12), where the rewrapping occurs on the milisecond time scale (23). This is much faster than our 50-ms time resolution, so we observe a constant FRET efficiency of about 0.8 in the absence of LexA (Figure 3A).

Upon the addition of LexA, we observe transient reductions in FRET efficiency to 0.2 (Figure 3A), the same FRET efficiency observed in ensemble measurements at saturating LexA concentrations (Supplementary Figure S4). The 200 s time series were acquired for >183 molecules at each LexA concentration (1.5, 5, 15 and 50 µM; Figure 3A). The dwell-time histograms of the nucleosome bound and unbound states were determined at each LexA concentration and fit to single exponential decays to determine the characteristic dwell times (Supplementary Figure S5A–H). The characteristic dwell time of the unbound state fit to *A*/[LexA] with an effective binding rate of *k*_on_ = *A*^−^^1 ^= (9 ± 2) × 10^−^^5^s^−1^ nM^−1^. The dwell time of the bound state was independent of LexA with a *τ*_bound_ = (0.31 ± 0.05) s implying an effective dissociation rate of *k*_off_ = 1/*τ*_bound_ = (3.3 ± 0.6)s^−1^ (Table 1).

During the smTIRF experiment, we confirmed the integrity of the nucleosome tethered on the microscope slide surface by examining the co-localization of FRET donor Cy3 and acceptor Cy5. Complete nucleosome complex showed Cy5 signal when excited with 638-nm laser, and either a high FRET if in the LexA unbound state or low FRET signal if in the bound state when excited with 532 nm. For each LexA concentration, 70% of the complete nucleosome molecules showed fluctuations in the FRET efficiency between the high- and low-FRET states. All fluctuating molecules were used in the dwell time histograms.

Comparison of the rates of LexA binding to and dissociating from duplex DNA and nucleosomes implies that the nucleosome reduces the binding rate by 500-fold and increases the dissociation rate by 1000-fold (Table 1). We determined the relative change in FRET from the single molecule and ensemble measurements (Figure 3C) and find they depend similarly on the LexA concentration indicating that the surface tethering does not significantly alter the TF binding and dissociation dynamics. We observe an increase of *S*_0.5_ by ∼10^5^ when LexA binds to the nucleosomes. This is consistent with our previous measurements of LexA binding to nucleosome with 130 mM NaCl (24) and is similar to RE measurements with nucleosomes containing a variant of the 601 sequence (22). Additional reports of TF binding within nucleosomes observe a smaller increase of *S*_0.5_ (11). However, these measurements were done in low-ionic conditions of ∼1 mM. We have previously reported that these changes in ionic conditions dramatically impact the TF concentration required to bind within nucleosomes (24).

We chose to label H2A because H2A–H2B heterodimers dissociate before the H3–H4 tetramer (36). Therefore, the fact that we observe significant energy transfer for each molecule implies that we are not detecting LexA binding to tetrasomes (DNA molecule only bound to the H3–H4 tetramer). In addition, we compared the Cy5-labeling efficiency of 0.88 that was measured by absorption spectrometry to the labeling efficiency predicted by the measured fraction of nucleosomes in the single-molecule FRET experiments with two Cy5 fluorophores relative to one Cy5 fluorophore. This allows for an estimate of the fraction of nucleosomes relative to hexisomes (nucleosomes that are missing one H2A–H2B dimer). We determined for 763 molecules the emission intensity of Cy5 during direct excitation by 638 nm, *I*_Cy5_direct,_ and the Cy5 emission intensity from the high-FRET-efficiency state, *I*_Cy5_FRET_, during excitation by 532 nm. The histogram of *I*_Cy5_direct_/*I*_Cy5_FRET_ has two distinct peaks, since each the molecule can contain either one or two Cy5 fluorophores (Supplementary Figure S5I). *I*_Cy5_direct_ was rescaled by *I*_Cy5_FRET_ to help remove the variation in emission intensity that is due to the spatial excitation variation of our smTIRF microscope. The histogram was fit to a sum of two Gaussian distributions. For nucleosome with full HO, the ratio of the areas under the Gaussian distributions are related to the labeling efficiency by *A*_two_Cy5_/*A*_one_Cy5_ = *L_P_*^2^/2*L_P_* (1 – *L_P_*), where *L_P_* is the labeling efficiency, and *A*_two_Cy5_ and *A*_one_Cy5_ are the areas under the Gaussian distributions for two and one Cy5 molecules, respectively. We measured the ratio of the areas to be 1.4, which implies a predicted labeling efficiency *L_P_* of 0.74. The similarity between the measured and predicted Cy5-labeling efficiencies indicates that most of the molecules measured are nucleosomes with a full HO.

### LexA binding and dissociation kinetics within dinucleosome arrays and mononucleosomes are similar

*In vivo*, nucleosomes are imbedded into long chromatin molecules. Therefore, we carried out similar LexA-binding experiments with dinucleosome arrays to determine if the presence of an adjacent nucleosome influences TF binding and/or dissociation (Figure 3A and B and Supplementary Figure S6). As with the previous measurements, we used ionic conditions of 130 mM NaCl and no divalent ions to mimic open euchromatin. We find that the LexA-binding and -dissociation rates to dinucleosomes are identical to mononucleosomes (Table 1). In addition, we determined the relative change in FRET from the single molecule and ensemble measurements with dinucleosome arrays (Figure 3C) and find they depend similarly on the LexA concentration indicating that the surface tethering does not significantly alter the TF binding and dissociation dynamics. These results suggest that within open euchromatin, a neighboring nucleosome does not impact TF binding and dissociation dynamics.

### Gal4 has a resident time much greater than 30 min at its consensus sequence within duplex DNA

We were concerned that the dramatic impact of the nucleosome on TF dissociation was unique to LexA. Furthermore, LexA is a prokaryotic TF and does not interact with nucleosomes *in vivo*. Therefore, we investigated the influence of nucleosomes on the binding and dissociation of the eukaryotic TF, Gal4. Gal4 is a model eukaryotic TF that recognizes its 19-bp consensus binding site with a *S*_0.5_ ∼ 10 pM (37), binds DNA as a homodimer (37,38), and upon binding activates the Gal1/10 genes in *S. cerevesiea* (39,40). As with previous studies, we used the first 147 amino acids of Gal4, which includes the DNA recognition and dimerization domains (37). While the Gal4 dissociation rate has not been reported, other TFs such as Glucocorticoid Receptor and NF-κB, which bind with picomolar dissociation constants, dissociate from duplex DNA on the hour time scale (13,14).

**Figure 4.**
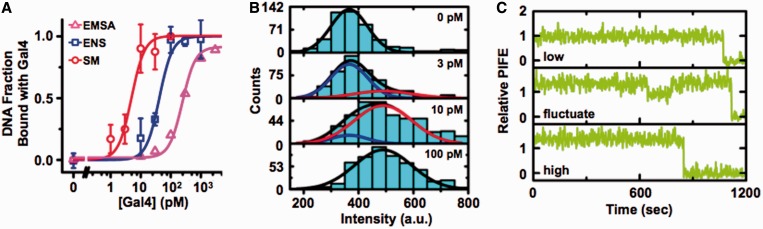
Single molecule measurements of Gal4 binding and dissociation to DNA. (A) The fraction of DNA bound by Gal4 with a cooperative binding curve fit as determined by EMSA [magenta triangles, *K_D_* = (240 ± 10) pM, Hill coefficient = 1.5], ensemble PIFE measurements [blue squares, *K_D_* = (42 ± 5) pM, Hill coefficient = 1.5] and the single molecule Cy3 fluorescence-intensity histograms from panel B [red circles, *K_D_* = (5 ± 1) pM, Hill coefficient = 2]. (B) Fluorescence distributions from Cy3-labeled DNA molecules containing the Gal4-target site with 0, 3, 10 and 100 pM Gal4 (ordered from top to bottom, respectively). The distributions were fit with the sum of two Gaussian distributions (black): the distribution without Gal4 (blue) and with 100 pM Gal4 (red). (C) Example Cy3 emission time traces of Cy3-labeled DNA containing the Gal4-recognition site without Gal4 bound (top), a dissociation and binding event (middle) and bound with Gal4 (bottom).

We used ensemble PIFE and EMSA to detect Gal4 binding to its recognition sequence within duplex DNA (Figure 4A, Supplementary Figure S3B). The EMSA measurements, which were done with 200 pM DNA, determined a *S*_0.5_ of (242 ± 10) pM, while the PIFE measurement, which were done with 100 pM DNA, determined a *S*_0.5_ of (42 ± 6) pM. Both methods determined a *S*_0.5_ that was similar to the concentration of the DNA used in the measurement, indicating that the dissociation constant *K_D_* is significantly less than 100 pM, which is consistent with previous measurements (37) and that PIFE can be used to detect Gal4 binding to its consensus site. Furthermore, we confirmed PIFE did not increase in the presence of Gal4 with Cy3-labeled DNA that did not contain a Gal4-target sequence (Supplementary Figure S1B). This demonstrates that PIFE is only detecting Gal4 binding to its target sequence.

We then carried out smTIRF measurements of Cy3–Cy5-labeled duplex DNA (Figure 1D) with the Gal4-recognition sequence. We acquired 600-s time series of Cy3 fluorescence at Gal4 concentrations of 0, 1, 3, 10, 30 and 100 pM for > 300 molecules at each concentration. We found that only a small fraction of DNA molecules displayed PIFE fluctuations. So we acquired 2000-s time series with 30 and 0 pM Gal4 (Figure 4C), and found that the fractions of time series with one or more PIFE fluctuations were (7 ± 2)% and (1.6 ± 0.6)%, respectively. To confirm that Gal4 is binding to its DNA-target sequence during the smTIRF experiments, we plotted histograms of the Cy3 fluorescence intensity from each Cy3–Cy5-labeled DNA molecule at each Gal4 concentration (Figure 4B) during the 600-s time series. We observe a shift in the fluorescence distribution at a concentration of ∼10 pM. We fit each fluorescent distribution as the sum of two Gaussian distributions: the distribution without Gal4, and the distribution with 100 pM Gal4. We determine the relative area under the high distribution to the area under the full distribution and plotted the relative change in area of the high peak and find it fits to a binding curve with a *S*_0.5_ of (5 ± 2) pM, which is similar to the previously report *K_D_* of 10 pM (37). This *S*_0.5_ value is also less than our EMSA and ensemble PIFE measurement as expected since these ensemble measurements determined a *S*_0.5_ similar to the concentration of the DNA. These results indicate that our smTIRF measurements are representative of Gal4 binding in solution. Our combined observations that most of the duplex DNA-target sites are bound by Gal4 at 30 pM, and that ∼90% of these bound molecules do not dissociate during the entire 2000-s acquisition implies that Gal4 remains bound to its site for much greater than 2000 s and that the dissociation rate is much less than 0.0005/s.

### Gal4 has a resident time of 50 s at its target sequence within the nucleosome entry–exit region

**Figure 5.**
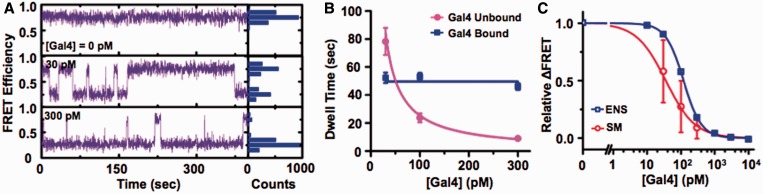
Single molecule measurements of Gal4 binding and dissociation to nucleosomes. (A) Single FRET traces of Gal4 trapping nucleosomes in partially unwrapped states with 0 (top), 30 (middle) and 300 (bottom) pM Gal4. The histogram shows the distribution of the FRET for each trace. (B) The unbound (magenta circles) and bound (blue squares) dwell times with single nucleosomes as a function of Gal4 concentration. Each dwell time was determined from an exponential fit to the dwell-time histogram (Supplementary Figure S7). The Gal4 concentration dependence of the unbound and bound dwell times were fit to *τ*_unbound_ = *A*/[Gal4] with *A* = (2.5 ± .01) s nM, while the bound dwell times were fit to *τ*_bound_ = constant = (50 ± 2) s. (C) The relative change in energy transfer efficiency from mononucleosome versus the Gal4 concentration was determined by both ensemble and single molecule measurements. The ensemble measurements relied on analysis of fluorescence spectra (Supplementary Figure S4) by the (ratio)_A_ method (blue squares). The relative change in FRET was determined from single-moelcule FRET time series (red circles) by determining the fraction of time each time series is in the low- and high-FRET states.

We investigated the influence of the nucleosome on Gal4 binding with smTIRF as we did with LexA binding to nucleosomes. We prepared sucrose gradient purified nucleosomes where the 20-bp LexA-recognition sequence was replaced with the 19-bp Gal4 consensus sequence. Changes in FRET efficiency were used to detect binding of Gal4 as it traps the nucleosome in a partially unwrapped state. We recorded 400 s time series of Cy3 and Cy5 fluorescence for >529 single nucleosomes at each Gal4 concentration (30, 100 and 300 nM). We observe fluctuations in FRET efficiency similar to the LexA measurements with nucleosomes (Fig. 5A). We quantified the dwell time of each bound and unbound nucleosome state, determined the dwell-time histogram for each Gal4 concentration, and fit each to a single exponential decay to determine the bound and unbound characteristic dwell times (Supplementary Figure S7). The characteristic dwell time of the unbound state fit to *A*/[Gal4] with an effective binding rate of *k*_on_ = *A*^−^^1 ^= (0.40 ± 0.02)s^−1^ nM^−1^ (Figure 5B and Table 1). The characteristic dwell time of the bound state was independent of Gal4 with a *τ*_bound_ = (50 ± 2) s, implying an effective dissociation rate of *k*_off_ = (0.020 ± 0.001)s^−1^ (Figure 5B and Table 1). This demonstrates that the Gal4-dissociation rate is at least 100-fold greater from nucleosomes relative to duplex DNA, confirming the LexA results. We determined the relative change in FRET for the single molecule and ensemble measurements (Figure 5C) and find they depend similarly on Gal4 concentration indicating that the surface tethering does not significantly alter the TF binding and dissociation dynamics.

## DISCUSSION

We find that the rate of TF binding to a target site within the nucleosome entry–exit region relative to duplex DNA is reduced by over two orders of magnitude while the rate of dissociation is enhanced by three orders of magnitude. The site exposure equilibrium, *K*_eq_, will impact the TF-binding rate since LexA can only bind to the unwrapped nucleosome state. Under the assumption that the LexA-binding rate to its site within duplex DNA is the same as to a site unwrapped from the nucleosome, the 500-fold reduction in the binding rate is equal to the reduction in probability that the target site is exposed. This regulation of TF binding by site exposure via transient unwrapping of nucleosomal DNA is well-established (12). However, the finding that nucleosomes also regulate TF occupancy by dramatically increasing the rate of protein dissociation appears to be a new observation. This provides an additional mechanism to regulate TF occupancy. Given that we observe a dramatic increase in dissociation rate for two separate TFs, this appears to a general feature of TF binding within nucleosomes. While regulating TF-binding rates will largely be influenced by nucleosome properties such as DNA sequence, histone PTMs and location of the target sequence (24,25,41), the regulation of TF dissociation rates will be influenced by both nucleosome and TF properties.

**Figure 6.**
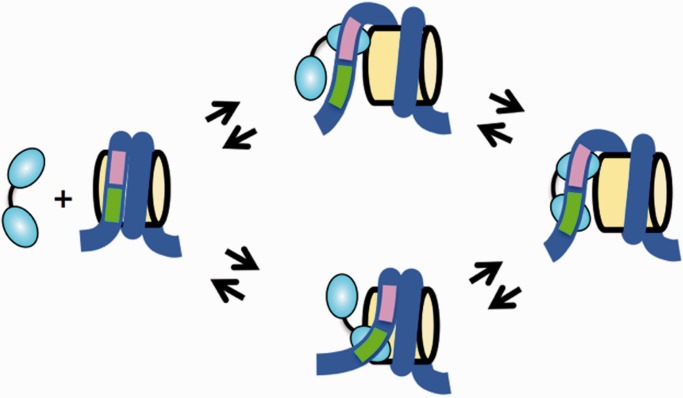
Model of competitive binding between nucleosome wrapping and TF binding. TFs could partially dissociate where part of the TF transiently releases from the DNA-target site and then rapidly fully rebinds again. However, if the site is located within the nucleosomes and the part of the TF further into the nucleosome transiently releases, than the nucleosome could rewrap preventing the TF from fully rebinding, which could increase the rate at which the TF fully dissociates.

There are at least two non-exclusive models by which the nucleosome could enhance TF dissociation. (i) The nucleosome could directly influence the TF-dissociation rates by altering the structure of the recognition site that is exposed for TF binding within the partially unwrapped nucleosome relative to the duplex DNA structure such that the TF resident time is shortened. This would be manifested by a direct change in *k*_off_ (Figure 1E). In addition, (ii) partially unwrapped nucleosome states could compete with partially bound TF states (Figure 6). For example, the LexA dimer binds with nanomolar affinities, while the LexA monomer binds with micromolar affinities (33), suggesting that the monomer has a much larger dissociation rate. If the inside portion of a TF dimer were to transiently dissociate, the nucleosome could partially rewrap blocking the rebinding of the inner portion of the TF. With only the outer monomer of the TF dimer bound, it is effectively bound as a monomer with a significantly increased dissociation rate. The partial rewrapping that blocks rebinding of the inner portion of the TF could result in a transient intermediate FRET state, which we do not observe. However, the lifetime of this state is likely to be on the scale for nucleosome rewrapping, which occurs on the millisecond time scale (23) and is too fast for us to detect. Future studies are required to determine the mechanism behind the increase in TF dissociation rate. Interestingly, the competitive model is similar to how adjacent TFs bind within nucleosomes cooperatively (42,43).

Our results also appear to be consistent with previous reports of competitive binding between high affinity DNA-binding proteins. A number of sequence independent DNA-binding proteins have resident times of ∼1 h (44,45) in the absence of soluble protein. However, upon the addition of soluble protein (45) or mechanical strain (46), the resident times are reduced to minutes. It was proposed that DNA-binding proteins undergo ‘micro dissociation’ where the protein partially or fully dissociates from the DNA but remains within the screening length (1 nm) of the DNA, and is much faster than the macroscopic dissociation rate (45). These rapid brief excursions of the bound protein from the DNA may allow soluble proteins to compete with rebinding thus increasing the macroscopic dissociation rate. Similarly, it appears that nucleosome rapid unwrapping and rewrapping could similarly function to compete with TFs that undergo micro dissociation to increase the dissociation rate.

While eukaryotic TFs can bind their target sequences with picomolar dissociation constants, they can be at nanomolar concentrations or higher within the cell (47,48). Under these conditions the placement of a nucleosome relative to the TF-target sequence will influence both the TF occupancy and dynamics. Gene promoters with TF-target sites positioned outside of a nucleosome will be occupied by the TF and remain bound for hours resulting in a constituently activated gene. Under these conditions, the dissociation rate is so slow that it will take on the order of the cell-cycle time to reach equilibrium. Therefore, the rate at which the TF can bind will determine the rate of gene activation, implying that it is kinetically controlled. Gene promoters with TF-target sites within the entry–exit region of the nucleosome will also be occupied by the TF at nanomolar concentrations, such as Gal4, but will exchange on the second to minute time scale, allowing for rapid regulation of the gene. Here, the equilibrium between the bound and the unbound state will determine the occupancy, which can be tuned by TF concentration. In contrast, gene promoters with TF-target sites near the nucleosome dyad symmetry axis will rarely be exposed and therefore will not likely be accessible for TF binding resulting in an inactive gene unless acted upon by chromatin modifying and remodeling complexes. Interestingly, bursts of TF localization and mRNA production has been reported to be on the minute time scale by single cell measurements (49–51), suggesting that the influence of nucleosomes on TF dissociation could play a regulatory role of transcriptional bursts.

Numerous other proteins bind DNA within nucleosomes including DNA repair and DNA replication complexes. The influence of the nucleosome on the binding and dissociation kinetics of other DNA-binding complexes may play a regulatory role of their functions. Future studies of the binding dynamics of other DNA-binding complexes to sites within chromatin will be important for determining if this feature of the nucleosome plays a regulatory role in other types of DNA processing.

## SUPPLEMENTARY DATA

Supplementary Data are available at NAR Online.

Supplementary Data
