# Supplementary material for: Nucleosomes accelerate transcription factor dissociation
Source: Nucleic Acids Res. 2013 Dec 17;42(5):3017–27. doi: 10.1093/nar/gkt1319 (PMC3950707; doi:10.1093/nar/gkt1319)
Supplement: Supplementary Data [file supp_42_5_3017__index.html]

Nucleosomes accelerate transcription factor dissociation — Supplementary Data 

# Nucleosomes accelerate transcription factor dissociation

## Supplementary Data

files

**Files in this Data Supplement:**

- Supplementary Data - pdf file
