# Supplementary material for: Nucleosomes accelerate transcription factor dissociation
Source: Nucleic Acids Res. 2013 Dec 17;42(5):3017–27. doi: 10.1093/nar/gkt1319 (PMC3950707; doi:10.1093/nar/gkt1319)
Supplement: Supplementary Data [file supp_gkt1319_nar-02794-m-2013-File008.pdf]

SUPPLEMENTARY FIGURES

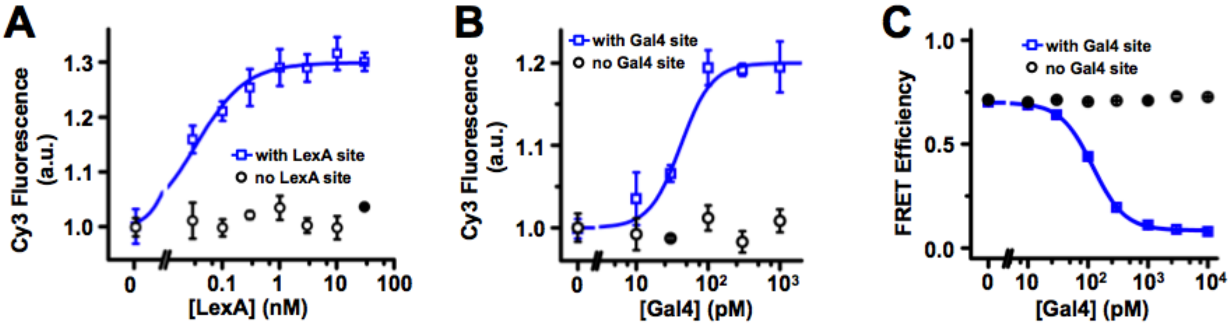

Supplementary Figure S1 Ensemble Cy3 fluorescence intensity measurements of (**A**) LexA and (**B**) Gal4 titrations with duplex DNA containing a Cy3 fluorophore adjacent to the TF target sequence (blue squares) or without the TF target sequence (black circles). The DNA molecules were labeled as shown in Fig. 1D and at a concentration of 0.2 nM for LexA titrations and 0.1 nM for Gal4 titrations. The fluorescence intensity was normalized to the average intensity in the absence of LexA or Gal4. We observe an increase in Cy3 fluorescence intensity of the DNA with the TF target site, while there is no increase in Cy3 fluorescence without the TF target site. This indicates that the increase Cy3 fluorescence is due only to specific binding to its target site and is not increased by nonspecific binding. (**C**) Ensemble FRET efficiency measurements of Gal4 titrations with Cy3-Cy5 labeled nucleosomes that contains the Gal4 target site (blue squares) or without the Gal4 target site (black circles). The nucleosomes were labeled as shown in Fig. 1E. We do not observe a Gal4 dependent decrease in FRET efficiency without the Gal4 site. This indicates that the reduction in FRET efficiency is due only to Gal4 binding specifically to its recognition site and is not reduced by nonspecific Gal4 binding.

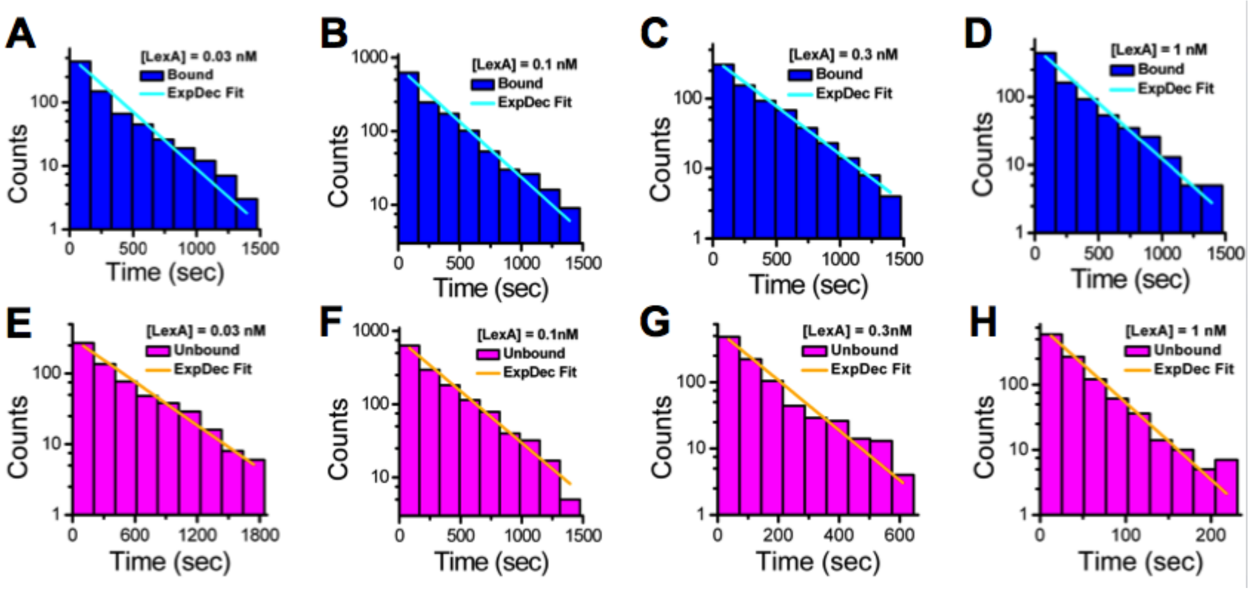

Supplementary Figure S2 (**A**-**D**) Dwell time histograms of duplex DNA bound with LexA at concentrations of 0.03, 0.1, 0.3 and 1 nM. (**E**-**H**) Dwell time histograms of duplex DNA without LexA bound at concentrations of 0.03, 0.1, 0.3 and 1 nM. Each histogram was fit to a single exponential decay to determine the characteristic dwell time, which is plotted in Fig. 2B.

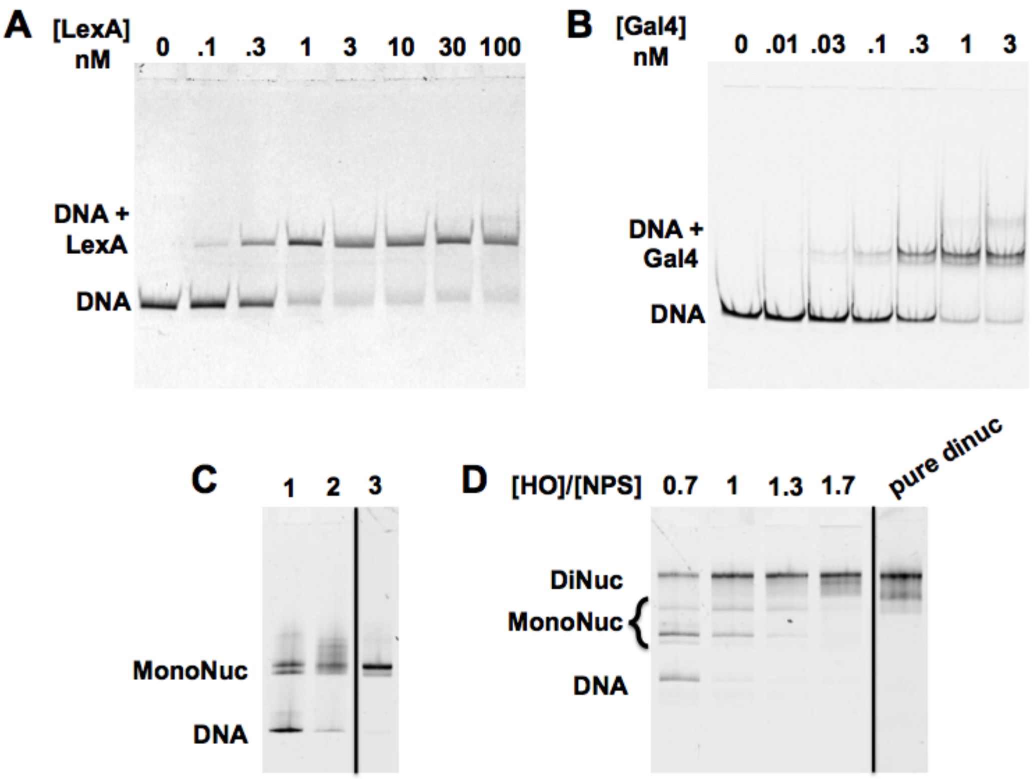

Supplementary Figure S3 (**A**) Electrophoretic Mobility Shift Assay (EMSA) of LexA binding to DNA. Each lane is labeled by the concentration of LexA. (**B**) EMSA of Gal4 binding to DNA. Each lane is labeled by the concentration of LexA. The quantification of the gels A and B are plotted in Fig. 2C and 4A, respectively. (**C**) Cy3 fluorescence image of EMSA of mononucleosomes with the LexA binding site before (lane 1), after sucrose gradient purification (lane 2) and with the Gal4 site after purification (lane3). (**D**) Cy3 fluorescence image of EMSA of dinucleosome arrays with increasing concentration of histone octamer (HO) to nucleosome positioning sequence (NPS). The lanes are labeled by the ratio of HO to NPS. The single molecule FRET experiments were done with sucrose gradient purified dinucleosome arrays prepared with a ratio of 1.3 (last lane).

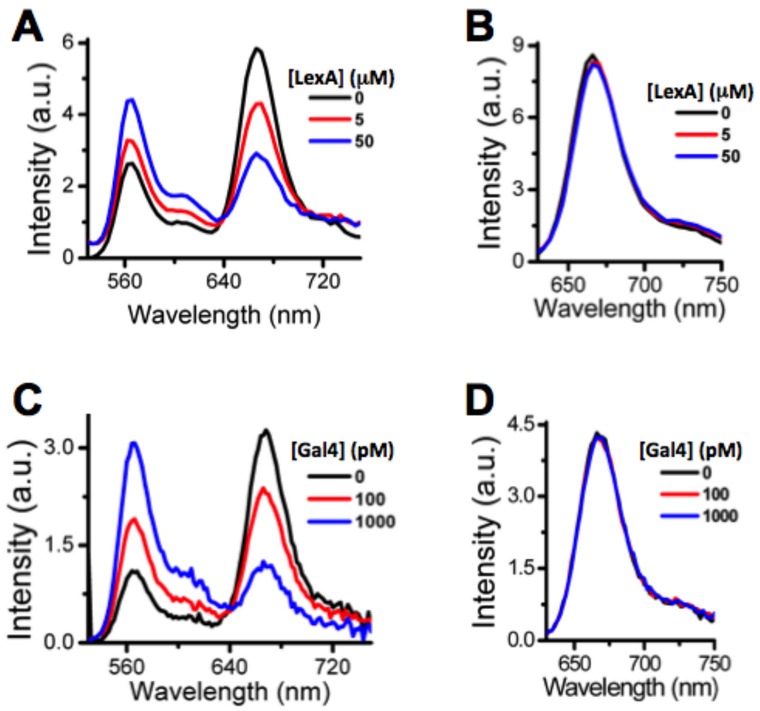

Supplementary Figure S4 (**A**) Fluorescence emission spectra at 0 nM (black), 5 μM (red), and 50 μM (blue) LexA, with nucleosomes containing the LexA target site being excited at 510 nm (donor excitation). (**B**) Fluorescence emission spectra at 0 nM (black), 5 μM (red), and 50 μM (blue) LexA, with nucleosomes containing the LexA target site being excited at 625 nm (acceptor excitation). (**C**) Fluorescence emission spectra at 0 pM (black), 100 pM (red), and 1000 pM (blue) Gal4, with nucleosomes containing the Gal4 target site being excited at 510 nm (donor excitation). (**D**) Fluorescence emission spectra at 0 nM (black), 100 pM (red), and 1000 pM (blue) Gal4, with nucleosomes containing the Gal4 target site being excited at 625 nm (acceptor excitation).

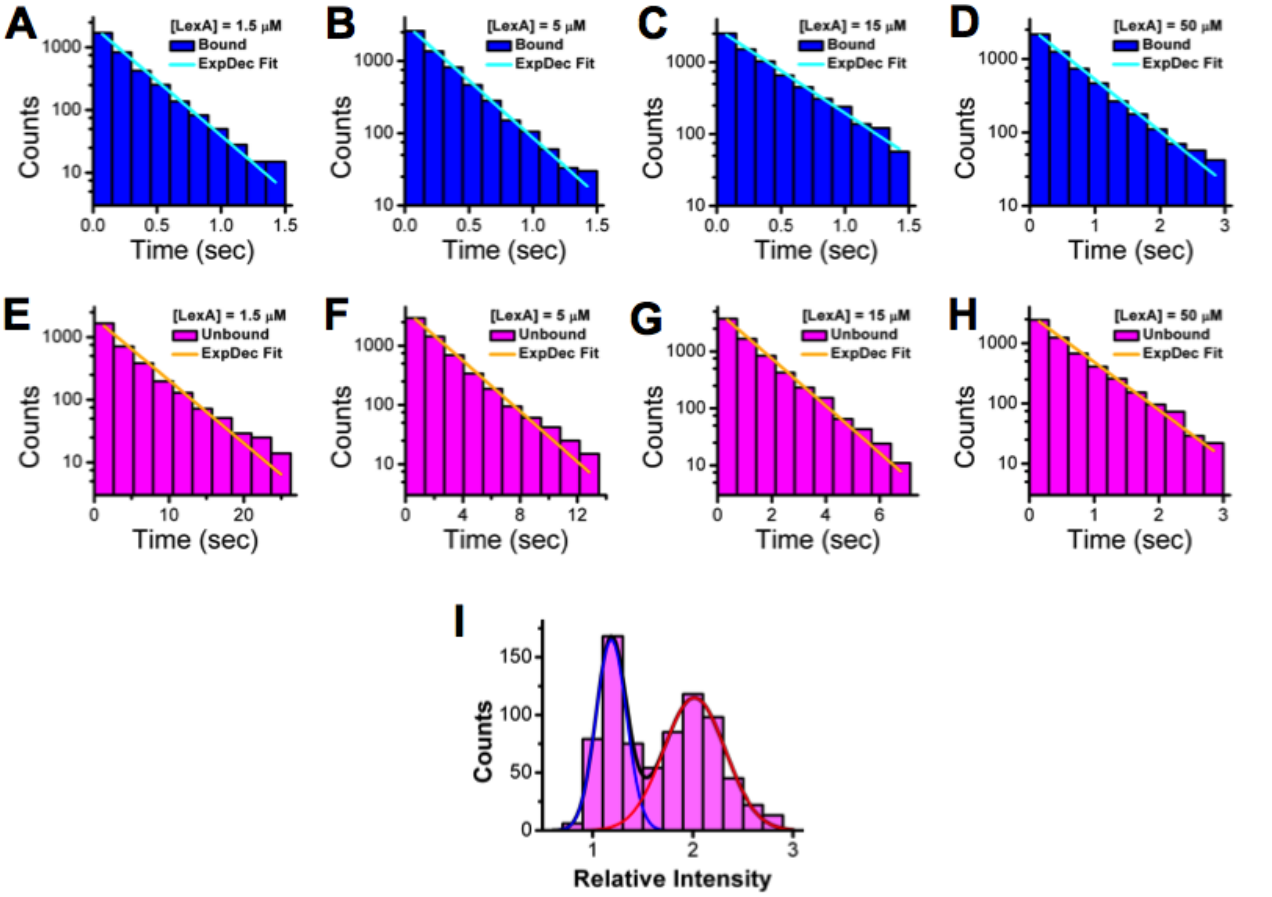

Supplementary Figure S5 (**A**-**D**) Dwell time histograms of partially unwrapped single nucleosomes bound with LexA at concentrations of 1.5, 5, 15 and 50 μM. (**E**-**H**) Dwell time histograms of single nucleosomes in the fully wrapped state without LexA bound at LexA concentrations of 1.5, 5, 15 and 50 μM. Each histogram was fit to a single exponential decay to determine the characteristic dwell time, which is plotted in Fig. 3B. (**I**) The histogram of the Cy5 fluorescence intensity while being directly excited at 638 nm. This was divided by the Cy5 fluorescence intensity while being excited by 532 nm to correct for spatial variation of the excitation of the smTIRF microscope. This distribution was fit to the sum of two Gaussian distributions. The ratio of the areas of the Gaussian distributions were used to determine the predicted labeling efficiency L_P 0.74 with A_{2_Cy5} / A_{1_Cy5} = L_p^2 / $2L_P(1-L_P)$. The measured labeling efficiency was measured by absorption spectrometry to be 0.88.

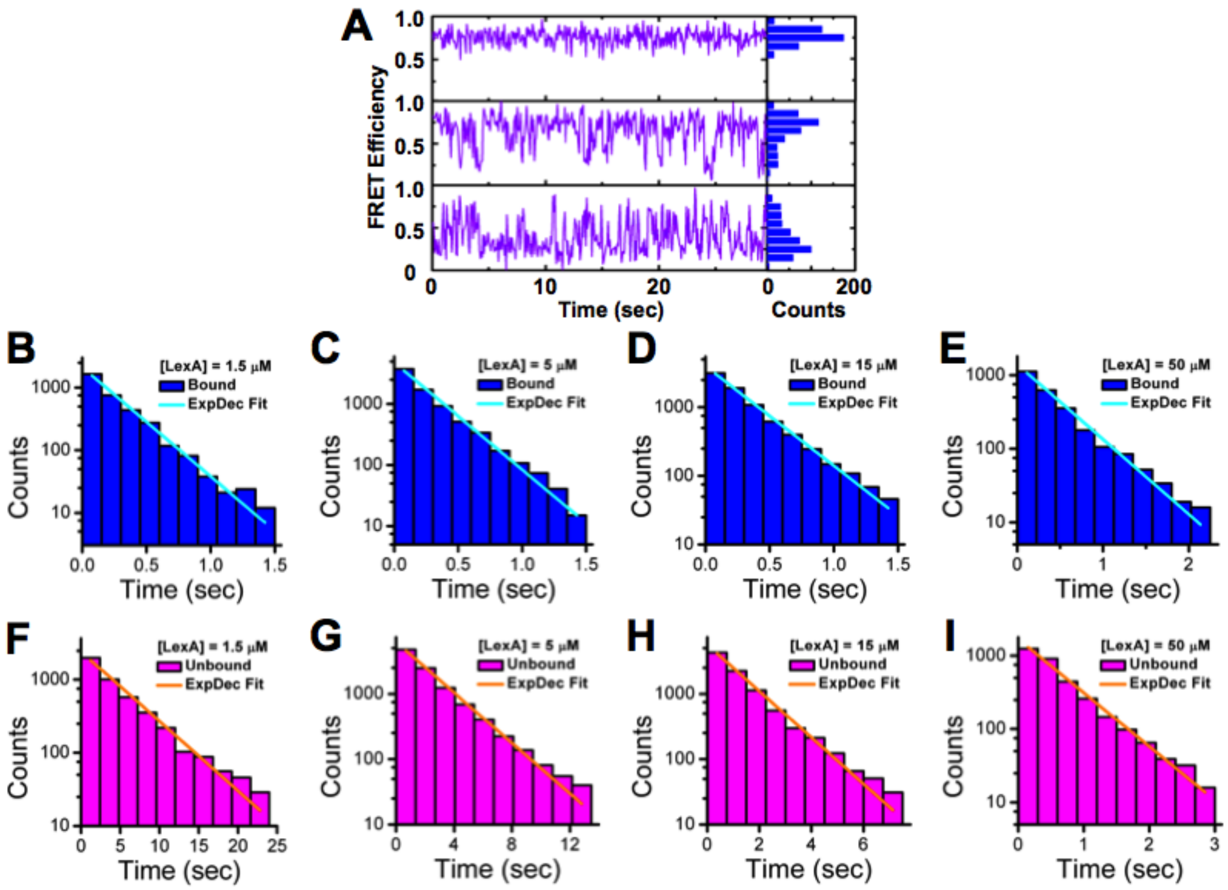

Supplementary Figure S6 (**A**) Single molecule FRET traces of LexA trapping a dinucleosome array in partially unwrapped states with 0 (top), 5 (middle), and 50 (bottom) μM LexA. The histogram shows the distribution of the FRET for each trace. (**B**-**E**) Dwell time histograms of partially unwrapped dinucleosome arrays bound with LexA at concentrations of 1.5, 5, 15 and 50 μM. (**F**-**I**) Dwell time histograms of dinucleosome arrays in the fully wrapped state without LexA bound at concentrations of 1.5, 5, 15 and 50 μM. Each histogram was fit to a single exponential decay to determine the characteristic dwell time, which is plotted in Fig. 3B.

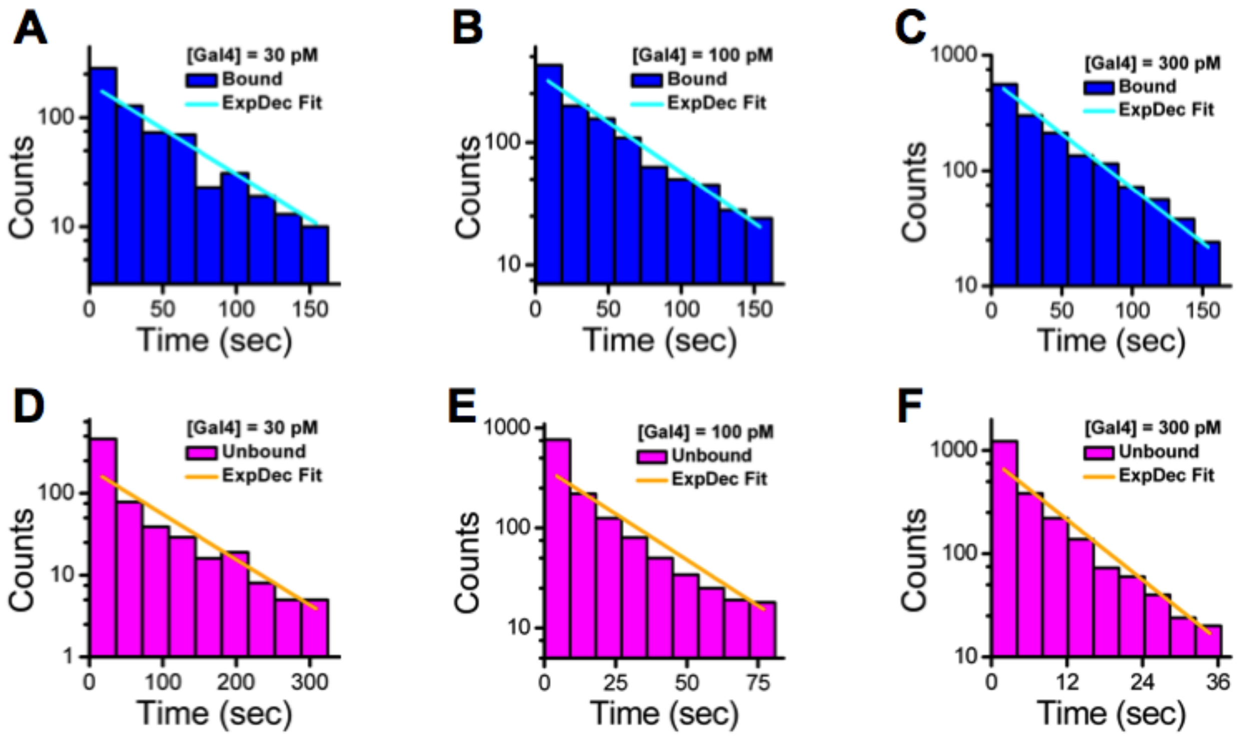

Supplementary Figure S7 (**A**-**C**) Dwell time histograms of partially unwrapped single nucleosomes bound with Gal4 at concentrations of 30, 100, 300 pM. (**D**-**F**) Dwell time histograms of nucleosomes in the fully wrapped state without Gal4 bound at concentrations of 30, 100, and 300 pM. Each histogram was fit to a single exponential decay to determine the characteristic dwell time, which is plotted in Fig. 5B.

DNA	Primer	Sequence
**dsDNA-LexA**	**forward**	aminoC6-5'-ATACTGTATGAGCATACAGTACAATTGGTCG TAGCAAGCT-3'
	**reverse**	biotin-5'-CCCCTTGGCGGTTAAAACG[aminoC6dT]GGGGG ACAGC-3'
**dsDNA-Gal4**	**forward**	5'-CTGGAG[aminoC6dT]CCGGAGGACTGTCCTCCGGTCAA TTGGTCGTAGACAGCTCTAGCACCGC-3'
	**reverse**	biotin-5'-CCCCTTGGCGGTTAAAACG[aminoC6dT]GGGGG ACAGC-3'
**nucDNA-LexA**	**forward**	aminoC6-5'-CTGGAGATACTGTATGAGCATACAGTACAATT GGTC-3'
	**reverse**	biotin-5'-CGCATGCTGCAGACGCGTT-3'
**nucDNA-Gal4**	**forward**	aminoC6-5'-CTGGAGACCGGAGGGCTGCCCTCCGGTC AATTGGTC-3'
	**reverse**	biotin-5'-CGCATGCTGCAGACGCGTT-3'
**dinucDNA-601**	**forward**	5'-CTGGAGAATCCCGGTGCCG-3'
	**reverse**	5'-CTAGCGTCAACCCAGTGTCACAGGATGTATATATCTG ACACGTGCCTGG-3'
**dinucDNA-601L**	**forward**	5'-AGCTTGTCGACGAATTCAGATCATAAGGAGGACACT GGGACATGCATCGGCTG[aminoC6dT]AGATACTGTAT-3'
	**reverse**	biotin-5'-CGCATGCTGCAGACGCGTT-3'

Supplementary Table S1 List of DNA oligos used for preparing the fluorophore labeled DNA molecules.
